# Laminin and Environmental Cues Act in the Inhibition of the Neuronal Differentiation of Enteric Glia *in vitro*

**DOI:** 10.3389/fnins.2019.00914

**Published:** 2019-09-03

**Authors:** Carla Pires Veríssimo, Juliana da Silva Carvalho, Fábio Jorge Moreira da Silva, Loraine Campanati, Vivaldo Moura-Neto, Juliana de Mattos Coelho-Aguiar

**Affiliations:** ^1^Instituto Estadual do Cérebro Paulo Niemeyer, Secretaria de Estado de Saúde do Rio de Janeiro, Rio de Janeiro, Brazil; ^2^Instituto de Ciências Biomédicas, Universidade Federal do Rio de Janeiro, Rio de Janeiro, Brazil; ^3^Pós-graduação em Anatomia Patológica, Faculdade de Medicina, Universidade Federal do Rio de Janeiro, Rio de Janeiro, Brazil

**Keywords:** enteric glia, neurogenesis, laminin, cell culture, microenvironment

## Abstract

The enteric glia, a neural crest-derived cell type that composes the Enteric Nervous System, is involved in controlling gut functions, including motility, gut permeability, and neuronal communication. Moreover this glial cell could to give rise to new neurons. It is believed that enteric neurons are generated up to 21 days postnatally; however, adult gut cells with glial characteristics can give rise to new enteric neurons under certain conditions. The factors that activate this capability of enteric glia to differentiate into neurons remain unknown. Here, we followed the progress of this neuronal differentiation and investigated this ability by challenging enteric glial cells with different culture conditions. We found that, *in vitro*, enteric glial cells from the gut of adult and neonate mice have a high capability to acquire neuronal markers and undergoing morphological changes. In a co-culture system with 3T3 fibroblasts, the number of glial cells expressing βIIItubulin decreased after 7 days. The effect of 3T3-conditioned medium on adult cells was not significant, and fewer enteric glial cells from neonate mice began the neurogenic process in this medium. Laminin, an extracellular matrix protein that is highly expressed by the niche of the enteric ganglia, seemed to have a large role in inhibiting the differentiation of enteric glia, at least in cells from the adult gut. Our results suggest that, in an *in vitro* approach that provides conditions more similar to those of enteric glial cells *in vivo*, these cells could, to some extent, retain their morphology and marker expression, with their neurogenic potential inhibited. Importantly, laminin seemed to inhibit differentiation of adult enteric glial cells. It is possible that the differentiation of enteric glia into neurons is related to severe changes in the microenvironment, leading to disruption of the basement membrane. In summary, our data indicated that the interaction between the enteric glial cells and their microenvironment molecules significantly affects the control of their behavior and functions.

## Introduction

The enteric nervous system (ENS) controls the motor, secretory and vascular functions of the gastrointestinal tract. The ENS is composed of neurons and enteric glial cells derived from the vagal and sacral neural crest. Enteric glial cells are involved in regulating the main functions of the gut, such as the regulation of the intestinal epithelial barrier, communication between neurons by modulating neurotransmission, and affect inflammation and many other gut diseases and alterations (Coelho-Aguiar Jde et al., 2015). The enteric glia is a highly plastic cell type, and the phenotype of these cells is dictated by cues from their specific location, i.e., intraganglionic, interganglionic, along nerve fibers in muscle layers, or in mucosa (Boesmans et al., 2015). Glia can assume a reactive phenotype in response to a specific stimulus, and can readily proliferate, both under steady-state conditions and after injury (Joseph et al., 2011).

Neural crest-derived progenitors exist in the adult peripheral nervous system (Dupin and Coelho-Aguiar, 2013). In the adult ENS, neural progenitors can give rise to neurons *in vitro* (Kruger et al., 2002; Bondurand et al., 2003) and in grafts to murine gut explants (Lindley et al., 2008; Metzger et al., 2009; Hetz et al., 2014). In *in vivo* experiments, Laranjeira et al. (2011) chemically destroyed ganglia with benzalkonium chloride (BAC) detergent and demonstrated the neurogenic capability of enteric glial cells in this specific damage condition. Enteric glial cell neurogenesis could also be induced *in vivo* through activation of the serotonin receptor (Liu et al., 2009). Some studies have shown that colitis can lead mouse and human enteric glial cells to undergo neurogenesis (Belkind-Gerson et al., 2015, 2017). Recently, Kulkarni et al. (2017) suggested that constitutive neurogenesis exists in the gut, although this study does not agree with data obtained by other groups that have investigated the matter (Pham et al., 1991; Young et al., 2003; Sasselli et al., 2012), which support the paradigm that intestinal neurons are stable and not easily replaced under healthy conditions. Moreover, the cited study evidences a population of nestin-positive adult progenitor cells that would be the source of these newborn neurons, different from that of GFAP-positive enteric glia, in contrast to previous work that had shown nestin and GFAP co-expression by enteric glial cells (Joseph et al., 2011).

It has been proposed that in cases of ganglion rupture and disruption of contact between cells (Gershon, 2011), such as in cell culture or in the chemical ganglion destruction with BAC detergent (Laranjeira et al., 2011), enteric glial cells have their neurogenic potential activated. However, this hypothesis has never been directly confirmed, and other factors may be involved. How would environmental changes be involved in this neurogenic differentiation of enteric glia?

One possibility is that changes in the extracellular matrix (ECM) in the ganglia niche can trigger the neuronal differentiation of enteric glia. The basement membranes of the muscle cells as well as that of the mucosal layer are rich in laminin, and the glial cells are located nearby. Enteric glia do not produce ECM proteins, but are surrounded by the basement membrane proteins including type IV collagen, laminin and a heparan sulfate proteoglycan (Bannerman et al., 1986; Neunlist et al., 2007). Previous investigations of laminin suggested that laminin-1 promotes migration of sox-10-positive enteric neural crest cells in mice (Nakazawa et al., 2013). Another study cultured neural progenitor cells from the adult rabbit jejunum on substrates composed by different combinations of ECM molecules, including laminin, heparin sulfate and collagen; and found that these molecules did not seem to inhibit the neuronal or glial fate after 5 and 15 days in culture (Raghavan et al., 2013). The composition of the ECM in engineered intestinal smooth-muscle sheets modulates the subtype of neurons differentiated from progenitor neural cells isolated from adult rabbit jejunum (Raghavan and Bitar, 2014). Moreover, a laminin-511 substrate enabled self-renewal in an undifferentiated state of other progenitor cell types, as cultured human embryonic stem cells and induced pluripotent stem cells (Domogatskaya et al., 2008; Miyazaki et al., 2008; Rodin et al., 2010). The engagement of postnatal hippocampal neural progenitor cells with a laminin substrate causes changes in the expression of connexin types and is associated with decreased neurogenesis of these cells in culture (Imbeault et al., 2009).

In spite of the known neurogenic potential of enteric glia, no study has addressed the question of how enteric glia are activated to differentiate in neurons. Here, we challenged enteric glial cells from adult and neonate mice with different cell culture conditions. We described the initial steps of neuronal differentiation of enteric glia in cell culture and investigated the role of the crosstalk between enteric neural cells and mesenchymal cells, in a co-culture with embryonic fibroblasts, as well as the role of the factors secreted by this fibroblasts lineage. Subsequently, we investigated the role of the main basement membrane protein, laminin. Our observations suggested that enteric glial cells in culture without the proper substrate were stimulated to initiate neuronal differentiation. Therefore, it seems that the proper contact of adult enteric glial cells with laminin plays a crucial role in inhibiting their potential for neuronal differentiation.

## Materials and Methods

### Animals

Newborn (P0 or P1) and adult (P90–P120) male Swiss mice were used. This research project was approved by the Animal Use Ethics Committee of the Centro de Ciências da Saúde-Universidade Federal do Rio de Janeiro (CCS-UFRJ) (protocol no. 129/16).

### Murine Enteric Neural Cells Culture

ENS cells were obtained from the final portion of the ileum (except the cecum) and the whole colon of adult mice in a culture of adult enteric glia, or the whole intestine of neonatal animals. We collected and thoroughly washed the tissue with phosphate-buffered saline solution (PBS) containing fungizone and penicillin/streptomycin, and removed the mesentery and mucosa with the aid of a stereoscopic microscope. The tissue containing the muscle layers and myenteric and submucous plexuses was cut into smaller pieces and incubated with the enzymes collagenase II (Gibco) and DNAse I (Sigma Chemical Co., St. Louis, MO, United States) for 1 h at 37°C. The tissue pieces were then mechanically dissociated and centrifuged twice (1200 rpm for 5 min). Dissociated cells were plated onto glass coverslips previously coated with poly-L-lysin (Sigma-Aldrich, St. Louis, MO, United States) in DMEM-F12 medium (Gibco, Carlsbad, CA, United States) containing glutamine (2 mM; Calbiochem, San Diego, CA, United States), sodium bicarbonate (3 mM; Merck, Gibbstown, NJ, United States), penicillin/streptomycin (0.5 mg/ml; Sigma-Aldrich, St. Louis, MO, United States), 10% fetal bovine serum (FBS) and 2% chicken embryo extract (CEE). Cultures were maintained at 37°C in a humidified chamber with 5% CO_2_, 95% air. In order to distribute the cells homogeneously, the cells were re-plated, either on the first (neonatal cells) or third (adult cells) day of culture. Cells were trypsinized and cultures were established by plating 1.5-2.0 × 10^4^ cells onto 24-well plates containing glass coverslips coated with poly-L-lysin, laminin, fibronectin or 3T3 feeder-layer substrates. The coating of glass coverslips with the different substrates and preparation of 3T3-conditioned medium (3T3-CM) are described in the following topics.

### ENS Cell Co-culture on NIH/3T3 Fibroblast Feeder-Layer

The NIH/3T3 cell lineage monolayer was prepared with 8 × 10^4^ cells per well of the 24-well plate. After 48 h, 1.5 × 10^4^ cells from the passage of enteric glial-cell cultures (from neonatal or adult mice) were plated on a 3T3 feeder-layer. These co-cultures were maintained for 4 (cells from adult mice) or 5 days (cells from neonate mice), fixed, and analyzed by immunofluorescence.

### Culture of ENS Cells With NIH/3T3 Fibroblast-Conditioned Medium

The NIH/3T3 cells (1.3 × 10^6^ cells) were plated into 25 cm^2^ culture flasks. After 48 h, the culture medium (as described for murine enteric neural cells culture) was conditioned for 24 h, centrifuged at 1500 rpm for 5 min to remove dead cells, and collected for culture of ENS cells.

### Preparation of Laminin and Fibronectin Substrates

The poly-laminin substrate (Sigma-Aldrich, St. Louis, MO, United States) was prepared in a concentration of 50 μg/ml, in acid buffer (pH = 4) containing 20 mM sodium acetate and 1 mM calcium chloride according to Freire et al. (2012). This poly-laminin substrate was prepared and added to the plate on the previous day. After at least 12 h at 37°C, the wells with laminin were washed three times with PBS. The ENS cells were then plated and maintained at 37°C and 5% CO_2_.

Fibronectin (Sigma-Aldrich, St. Louis, MO, United States) was prepared in a concentration of 50 μg/mL in DMEM-F12 culture medium, added to the plate for 1 h at 37°C and then removed from the wells, which were allowed to dry. Then, the ENS cells were plated and maintained at 37°C and 5% CO_2_.

### Preparation of Frozen Histological Sections

The intestine sample obtained was fixed with 4% formaldehyde for 16 h at 4°C. After washing with PBS, the sample was incubated in 30% sucrose (diluted in PBS) for at least 16 h. The sample was then transferred to an OCT (Tissue-Tek, Sakura Finetek, United States) mold, and rapidly frozen by immersion in liquid nitrogen. We set the Leica CM1860 cryostat to a temperature of −20°C and made 15-μm sections of the frozen block. The sections were transferred to histology slides previously treated with poly-L-lysine (Sigma-Aldrich, St. Louis, MO, United States).

### Immunocytochemistry and Immunofluorescence in Histological Slides

For cells in culture (fixed in 4% paraformaldehyde for 5 min) or histological sections, the material was permeabilized with 0.2% Triton X-100 for 5 min at room temperature, and unspecific sites were blocked with 5% bovine serum albumin (Sigma) for 1 h before immunoreactions with the following primary antibodies: rabbit anti-GFAP (1:400; DAKO Cytomation), rabbit anti-S100β (1:100; Dako Cytomation), mouse anti-Sox10 (1:50; Santa Cruz Biotechnology), mouse anti-p75 (1:60; Millipore); mouse anti-βIIItubulin (1:500; Promega), mouse anti-HuC/D (1:50; Molecular Probes), rabbit anti-Peripherin (1:500; Millipore), mouse anti-smooth muscle actin (SMA) (1:400; Sigma), rabbit anti-Ki67 (1:200; BD pharmigen), rabbit anti-laminin (1:100; Sigma-Aldrich), rabbit anti-fibronectin (1:100; Sigma-Aldrich). After incubation with the primary antibody, the material was thoroughly washed with PBS and incubated with secondary antibodies for 1 h at room temperature. Secondary antibodies were: goat anti-mouse Alexa Fluor 546 (1:500) or goat anti-rabbit Alexa Fluor 488 (1:500) (Molecular Probes). Nuclei were counterstained with DAPI (4′,6-diamidino-2-phenyindole, dilactate; Sigma-Aldrich). The images were obtained with a Leica DMi8 inverted fluorescent microscope. Deconvolution of the images and Z projection analysis were performed. The images were obtained with the projection of 5 or more photos along the *Z* axis.

### Immunofluorescence *in toto*

The longitudinal muscle layer of the colon or ileum was dissected using a stereoscope microscope, and then the tissue containing the myenteric plexus adhered to the muscle tunica was fixed in 4% formaldehyde for 16 h at 4°C. The material was then permeabilized and blocked by incubating with 0.2% triton-X100 and 5% BSA for 1 h, and after several washes with PBS, was incubated with the primary antibody for 16 h at 4°C. Specific antibodies were used for glial (GFAP and S100β), neuronal proteins (βIIItubulin, Peripherin and HuC/D), and for laminin at the same dilution as in the immunocytochemistry protocol. Then, after washes with PBS, the material was incubated with the specific secondary antibody for 2 h at room temperature. Nuclei were counterstained with DAPI and the tissues mounted as histological sections. The images were obtained with a Leica DMi8 inverted fluorescent microscope.

### RT-PCR Experiments

RNA was extracted from the cultured cells using TrizolVR (Invitrogen), according to the manufacturer’s instructions. Complementary DNAs (cDNAs) were synthesized using a High-Capacity cDNA Reverse Transcription kit (Applied Biosystems) according to the supplier’s instructions, and were used as templates for the polymerase chain reaction (PCR). Primers were designed and synthesized by Sigma or IDT. Reverse and forward specific oligonucleotides were: GFAP: (F) TGC AAG AGA CAG AGG AGT GG, (R) CTC CAG ATC GCA GGT CAA GG; βIIItubulin: (F) CCC AGC GGC AAC TAT GTA GGG, (R) CCA GGT TCC AAG TCC ACC AGA A; P75: (F) CCA ACC AGA CCG TGT GTG AA, (R) CAC AGG GAG CGG ACA TAC TC; Sox10: (F) GCT GGA CCG CAC ACC TTG, (R) TCC TCG TGA AGA GCC CAA CG; Synaptophysin: (F) GTG TTT GCC TTC CTC TAC TC, (R) CAC ATA GGC ATC TCC TTG ATA A; Psd95: (F) TGC CAG ATG GAC AAG GAG ACC AAA, (R) TGT TGG CCT TGA GGT GGT AGA GTT; β-actin: (F) TGG ATC GGT TCC ATC CTG G, (R) GCA GCTCAG TAA CAG TCC GCC TAG A. PCR parameters were 94°C for 30 s (first cycle 2 min) for the denaturing step, 60°C for 30 s for the annealing step, and 72°C for 60 s for the elongation step, with a total number of 35 cycles. PCR included beta-actin as a control and reaction in the absence of the cDNA prepared in the reverse transcriptase reaction step (RT−).

### Statistical Analysis

GraphPad Prism version 5.0 (GraphPad Software, La Jolla, CA, United States) was used for statistical analysis of quantitative data. An Analysis of Variance (ANOVA) was followed by a Bonferroni post-test for multiple comparisons, and a *t*-test was performed for each pair of means. The results represent the mean of the quantification of three independent experiments. In each experiment, cells from 10 or more photos (using the 20x magnification objective) from different fields of the cell culture were quantified. A value of *P* < 0.05 was considered statistically significant. The data are reported as mean ± standard deviation; the error bars in the graphs represent the standard deviation.

## Results

### Enteric Glial Cells Migrate to Each Other and Acquire Neuronal Markers When Cultured *in vitro*

**FIGURE 1:**
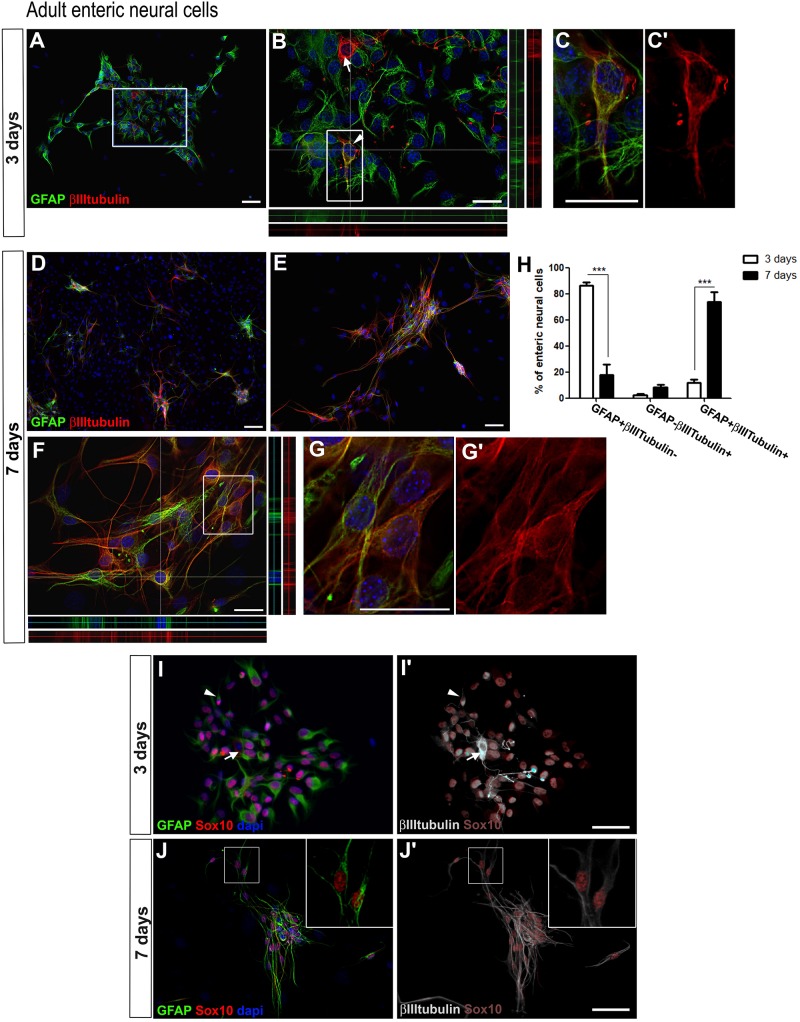
Adult enteric glial cells acquire βIIItubulin expression *in vitro*. **(A–G’)** Enteric neural cells immunostained for GFAP and βIIItubulin. **(A–C’)** At day 3, most of the cells were GFAP-positive. βIIItubulin expression was observed in neurons arising to the cell culture directly from intestinal tissue (arrow in **B**) or in few cells that were also positive for GFAP (arrowhead in **B**). **(B)** Higher magnification of selected field in **(A)**. Orthogonal view confirms the expression of GFAP, βIIItubulin or both at a given point. **(C,C’)** Double-labeled cell depicted in **(B)** (arrowhead) shown in higher magnification. **(D–G’)** At day 7, after replating than at day 3, most of the neural cells were organized in groups **(D,E)**, and most enteric neural cells showed expression of both GFAP and βIIItubulin. **(F)** Orthogonal view confirms the expression of GFAP, βIIItubulin or both at a given point. **(G,G’)** Double-labeled cells depicted in **(F)** shown in higher magnification. **(H)** Quantification of GFAP and/or βIIItubulin-expressing adult enteric neural cells at days 3 and 7 in cell culture. Cells expressing only GFAP showed a significant reduction from day 3 to day 7. Instead, the number of double-labeled cells increased markedly over the period. The results represent the mean of three independent experiments. ^∗∗∗^*P* < 0.001. **(I–J’)** Sox10 expression by cultured adult enteric nervous system cells at days 3 and 7. Same field is shown in **(I,I’)**. Same field is shown in **(J,J’)**. Most glial cells showed GFAP and Sox10 expression, but some expressed only Sox10 or GFAP. At day 3, βIIItubulin expression **(I’)** was rarely observed in neurons arising to the cell culture directly from intestinal tissue (arrow) or in cells that were also positive for GFAP and Sox10 (arrowhead). At day 7, most neural cells were labeled for both the glial markers and βIIItubulin. Two GFAP+Sox10+βIIItubulin+ cells are shown in higher magnification **(J,J’)**. Scale bars: 100 μm **(D,I–J’)**, 50 μm **(A,E)**, and 25 μm **(B,C’,F,G’)**.

Cells of the enteric nervous system were obtained from mouse colon and ileum, from dissociated tissue containing the myenteric and submucous plexus after mucosa removal. After 3 days, the cultured cells were analyzed (Figures 1A–C’), or they were harvested, individualized and re-plated. In these conditions, after re-plating, enteric neural cells proliferated and tended to organize themselves in groups (Figure 1D).

In addition to enteric neural cells, the target of this study, we found many cells that were negative for the neural markers at all times analyzed. Most of them were fibroblasts and expressed SMA protein (Supplementary Figure S1). After 3 days in cultures, neural cells accounted for 51.1–73.5%, and after 7 days they represented 29.4–48.9%. We represented in the graphics only the neural cells stained for the analyzed markers.

To evaluate the presence of enteric glial and neuronal cells, we analyzed the expression of the glial marker GFAP, and of βIIItubulin, a marker of early neuroblasts and mature neurons, at different culture times. In these cultures of ENS cells from adult mice, many GFAP-positive cells were detected at day 3 (Figures 1A,B). Cells positive for GFAP+βIIItubulin+ comprised 86.1 ± 4.3%, and only 11.6 ± 5.0% of the neural cells consisted of cells double-labeled with both the glial and neuronal markers. GFAP-βIIItubulin+ cells comprised only 2.2 ± 1.5% (Figure 1H). At day 7 (Figures 1D–G), 73.8 ± 12.9% of the neural cells co-expressed GFAP and the neuronal marker βIIItubulin (Figures 1G,G’), either isolated or in groups. Cells that expressed only GFAP comprised 17.6 ± 14.0%. Relatively few cells (about 8.4 ± 3.3%) expressed only the neuronal marker and correspond to neurons arising to the cell culture directly from intestinal tissue, that do not appear from the differentiation *in vitro* of enteric glial cells. Neurons obtained directly from the gut tissue were easily identified morphologically and had a clearly higher fluorescence intensity of βIIItubulin staining (arrow in Figure 1B, for example), compared to the GFAP and βIIItubulin double-labeled cells. They correspond to the GFAP-βIIItubulin+ cells found at days 3 and 7 of culture.

These initial results suggest that GFAP-positive enteric glial cells are in the initial steps of directly convertion into neurons *in vitro*, and co-expressed both markers. To better characterize this event, we investigated other glial markers. We found that most of the GFAP-positive cells also expressed Sox10 (Figures 1I–J’) and nestin (data not shown), and few cells expressed only one of these markers. Even at day 7, Sox10 was expressed by most of the GFAP-positive cells, which were also labeled with anti-βIIItubulin (Figures 1J,J’).

**FIGURE 2:**
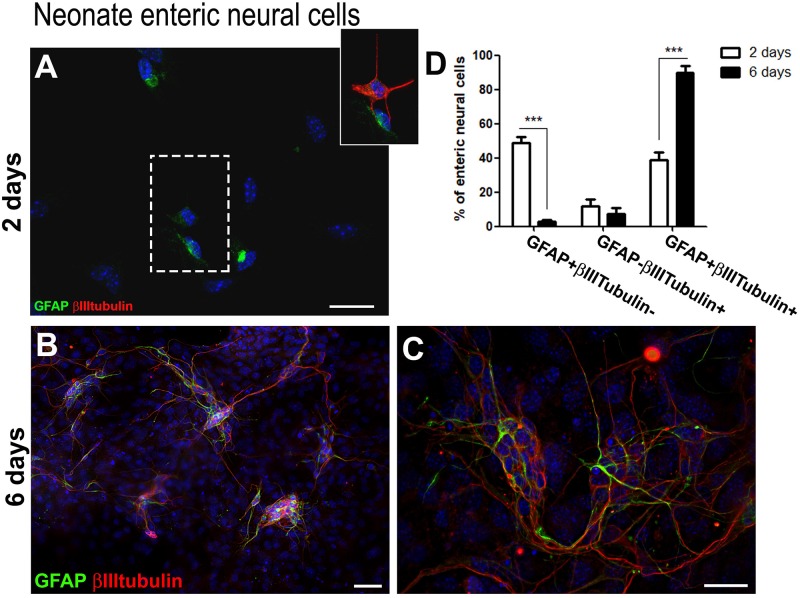
Neonate enteric glial cells acquire βIIItubulin expression *in vitro*. **(A–C)** Enteric neural cells immunostained for GFAP and βIIItubulin. **(A)** At day 2, a great proportion of GFAP-positive cells already expressed βIIItubulin. βIIItubulin expression was also observed in some neurons arising to the cell culture directly from intestinal tissue. The selected field, in higher magnification, shows a cell expressing only GFAP and other expressing both markers. **(B,C)** At day 6, most of the neural cells were organized in groups after replating than at day 1, and most enteric neural cells expressed both GFAP and βIIItubulin. **(D)** Quantification of numbers of GFAP and/or βIIItubulin-expressing neonate enteric neural cells at days 2 and 6 in cell culture. As occurred with the adult cells, neonate cells expressing only GFAP showed a significant reduction from day 2 to day 6. Instead, the number of double-labeled cells increased markedly over the period. The results represent the mean of three independent experiments. ^∗∗∗^*P* < 0.001. Scale bars: 50 μm **(B)** and 25 μm **(A,C)**.

This acquisition of the neuronal marker by enteric glia was also observed in cells from the neonate gut. Figure 2D shows the quantification of enteric neural cells expressing glial and neuronal markers after 2 and 6 days in culture.

We also obtained in culture of neonate enteric neural cells many negative cells for the neural markers. After 2 days in cultures, neural cells accounted for 14.4–18.8%, and after 6 days they represented only 14.4–21.2%. We represented in the graphics only the neural cells stained for the analyzed markers.

After 2 days (Figure 2A), most of the neural cells were enteric glia (GFAP + βIIItubulin−) (49.0 ± 6.0%), and 38.9 ± 7.3% were double-labeled. At day 6 (Figures 2B,C), 89.7 ± 7.4% of the cells co-expressed both GFAP and βIIItubulin. Once again, after replating the neural cells migrated to maintain contact with each other and were mainly organized in groups (Figure 2D). GFAP-βIIItubulin + cells represent a small percentage after 2 and 6 days.

**FIGURE 3:**
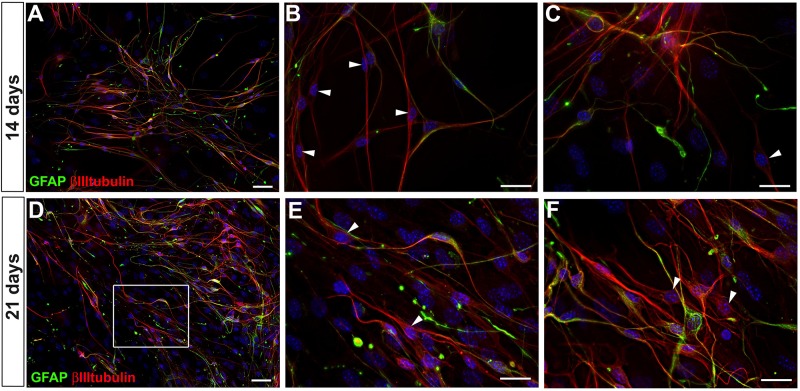
GFAP and βIIItubulin expression by adult mouse enteric nervous system cells at days 14 and 21 in culture. **(A–C)** Culture at day 14. **(D–F)** Culture at day 21. Stronger expression of βIIItubulin per cell. Cells expressing only βIIItubulin indicated by arrowheads in **(B,C,E,F)**. Scale bars: 50 μm **(A,D)** and 25 μm **(B,C,E,F)**.

After 14 and 21 days of adult enteric neural cells culture, neural cells that expressed both GFAP and βIIItubulin still predominated (Figure 3). However, βIIItubulin expression had a generally higher fluorescence level in most of positive cells. After 21 days in culture, the cells were replated three times, and we could not identify neurons arising to the cell culture directly from intestinal tissue, that do not appear from the differentiation *in vitro* of enteric glial cells (neither by the high expression of βIIItubulin nor by morphology), probably because of the culture passage procedures. Nevertheless, at days 14 and 21, GFAP-βIIItubulin+ cells were easily found (arrowheads in Figures 3B,C,E,F), suggesting that they had lost the GFAP expression in the neurogenic differentiation process. In fact, it was difficult to find a cell expressing GFAP that did not also express βIIItubulin. Even those with strong GFAP expression and glia morphology had at least a basal expression of βIIItubulin.

**FIGURE 4:**
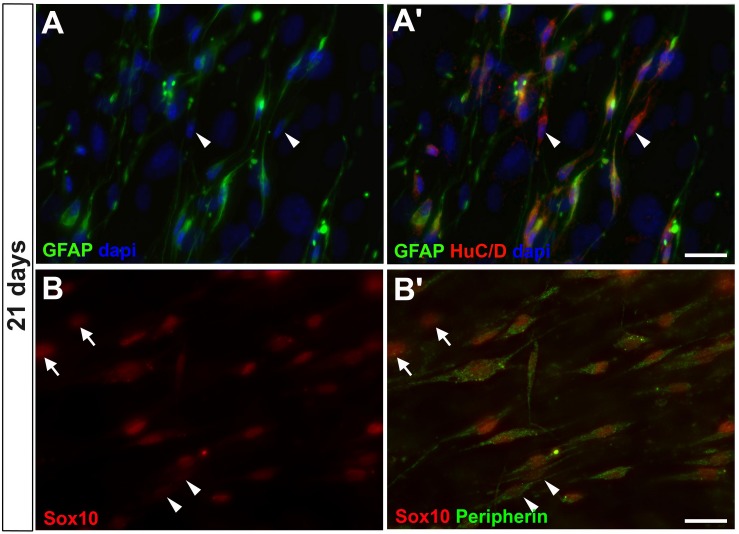
Enteric glial cells undergoing neurogenesis *in vitro* expressed HuC/D and peripherin after 21 days. **(A,A’)** Same field is shown in **(A,A’)**. Image shows cells expressing GFAP and HuC/D and some expressing only HuC/D (arrowheads). **(B,B’)** Same field is shown in **(B,B’)**. Cells expressing Sox10 and peripherin. Some of them expressed only peripherin (arrowheads), and others did not yet express peripherin, only Sox10 (arrows). Scale bars: 25 μm.

To investigate whether enteric glial GFAP-positive cells could progress toward acquiring neuronal markers, the cultures were further investigated for the presence of other mature neuronal markers at 7, 14 (data not shown) and 21 days (Figure 4). HuC/D, the RNA-binding protein that is present in immature and mature neurons, and Peripherin, an intermediate filament of mature neurons, could be observed at 7 days in only a few GFAP-negative cells (data not shown), corresponding to neurons arising directly from intestinal tissue. In cells cultured for 21 days, HuC/D was detected with a weak fluorescence intensity in GFAP-positive cells and some GFAP-negative cells (arrowheads in Figures 4A,A’). Peripherin was weakly expressed in most of Sox10-positive cells.

**FIGURE 5:**
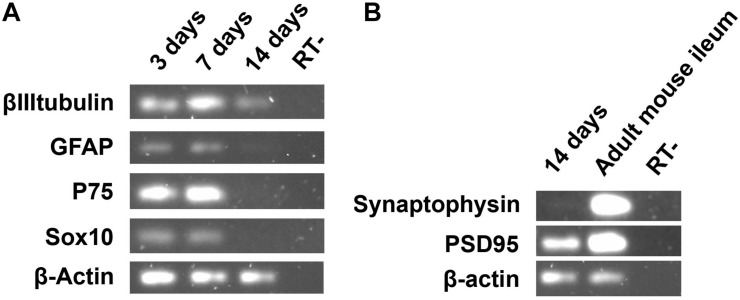
Semi-quantitative RT-PCR analysis of adult ENS cells in culture at days 3, 7, and 14. **(A)** The expression of βIIItubulin, neuron marker gene, and glial marker genes were evaluated. **(B)** The synaptic protein Psd95 was highly expressed after 14 days of enteric glial cell culture.

The *in vitro* development of enteric glia in the course of the neuronal fate was further characterized by semi-quantitative RT-PCR of cultures harvested from day 3 to day 14 (Figure 5). Data from the RT-PCR experiments confirmed the different levels of genes expressed by ENS cells over the course of the cell culture, as observed through immunocytochemical experiments. The level of βIIItubulin mRNA expression increased noticeably from 3 to 7 days, although the βactin level was slightly lower at 7 days, and even with the number of non-neural cells having grown considerably in this period, as mentioned before. At 14 days, βIIItubulin was notably higher than GFAP, which showed a basal expression (the low expression of GFAP at 14 days was probably due to a larger number of non-neural cells that proliferated from days 7 to 14). Sox10 and p75 showed similar levels of expression between days 3 and 7, and, similarly to GFAP, almost no expression after day 14, indicating the presence of βIIItubulin-positive cells that had lost their glial marker expression, as also observed by immunocytochemistry.

In addition to the glial and neuronal markers, we investigated the presence of a synaptic gene expression in our ENS cell cultures (Figure 5B). At day 14 we found no expression of synaptophysin, a protein that is usually present in neuronal synaptic density. Curiously, we observed expression of psd95, a protein involved in the organization of postsynaptic density, suggesting the development of neuronal activity of the glial cells that were becoming neurons.

Taken together, these data suggest that cultured enteric glia can undergo a process of neuronal differentiation.

### Enteric GFAP-Positive Cells Acquire βIIItubulin Expression *in vitro* Independently of Their Proliferative State

**FIGURE 6:**
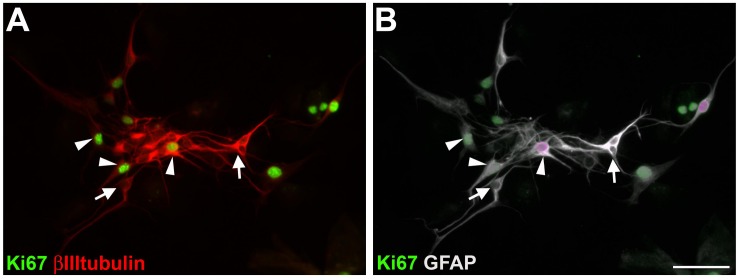
Proliferation of cultured adult mouse enteric nervous system cells. After 7 days *in vitro*, cells were immunostained for Ki67, βIIItubulin **(A)** and GFAP **(B)**. Same field is shown in **(A,B)**. GFAP and βIIItubulin double-labeled cells with (arrowheads) or without (arrows) Ki67 staining. Scale bar: 50 μm.

We analyzed the proliferative state of adult enteric neural cells cultured for 7 days (Figure 6). In our culture conditions, we observed many neural and non-neural proliferative cells. Double-labeled cells for GFAP and βIIItubulin could be in a proliferative state (arrowheads) or not (arrows). These results are consistent with a transdifferentiation process, since we observed βIIItubulin expression in most of the cells that were positive for GFAP and Sox10 (Figures 1I–J’).

### *In vivo* Enteric Glial Cells Do Not Express βIIItubulin

**FIGURE 7:**
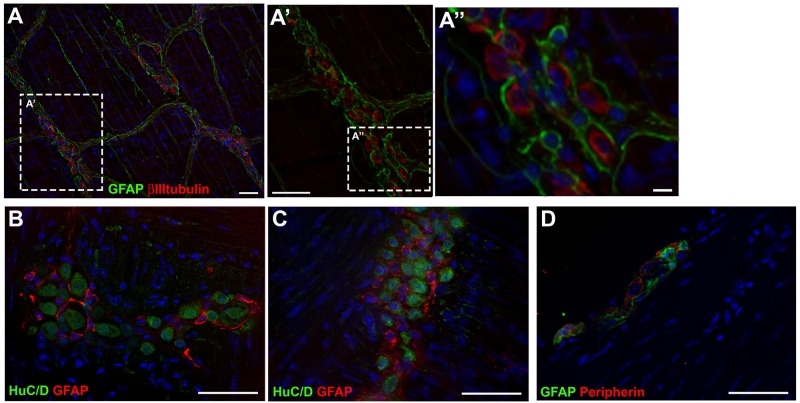
Expression of glial and neuronal markers in mouse gut tissue. **(A,A’)** Longitudinal muscle with the adherent myenteric plexus (LMMP) of adult mouse colon stained for GFAP and βIIItubulin. **(B,C)** LMMP of adult mouse ileum stained for GFAP and HuC/D. **(D)** Transverse section of mouse colon stained for GFAP and Peripherin. Scale bars: 25 μm **(A”)** and 50 μm **(A,A’,B–D)**.

Histological stains were used to evaluate the expression of the glial marker GFAP and the neuronal markers βIIItubulin, HuC/D and Peripherin, directly in the tissue. We observed that glial and neuronal markers did not usually co-localize. In the longitudinal muscle with the adherent myenteric plexus (LMMP) preparations (Figures 7A–C), GFAP did not co-localize with βIIItubulin (Figures 7A–A”) or with HuC/D (Figures 7B,C). In transverse sections of adult mouse colon, GFAP did not co-localize with peripherin (Figure 7D).

### Co-culture With Embryonic Fibroblasts Limits the Neuronal Differentiation Process of Enteric Glia

**FIGURE 8:**
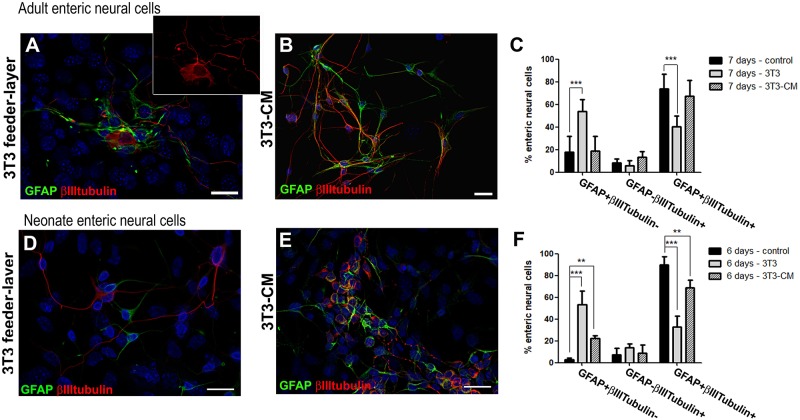
Culture of enteric glial cells on 3T3 fibroblast feeder-layer inhibits neurogenesis. Analysis by immunostaining for GFAP and βIIItubulin. **(A–C)** After cell passage on day 3, the adult enteric neural cells were maintained for more 4 days on 3T3 feeder-layer **(A)** or with 3T3-CM **(B)**. Quantification **(C)** of GFAP and/or βIIItubulin expressing adult enteric neural cells in control, on 3T3-feeder-layer, or with 3T3-CM treatment. Significantly fewer cells expressed both GFAP and βIIItubulin when cultured on 3T3 feeder-layer. The results represent the mean of 3 independent experiments. ^∗∗∗^*P* < 0.001. **(D–F)** After cell passage on day 1, neonate enteric neural cells were maintained for 5 more days on 3T3 feeder-layer **(D)** or with 3T3-CM **(E)**. Quantification **(F)** of GFAP and/or βIIItubulin expressing neonate enteric neural cells in control, on 3T3 feeder-layer, or with 3T3-CM treatment. Cells expressing only GFAP significantly increased in the 2 new conditions. The number of double-labeled cells showed a significant reduction with 3T3-CM and even more on the 3T3 feeder-layer. The results represent the mean of 3 independent experiments. ^∗∗^*P* < 0.01, ^∗∗∗^*P* < 0.001. Scale bars: 25 μm.

*In vivo*, the enteric neural cells are influenced by different secreted molecules and in contact with specific substrates, influenced by muscle, fibroblastic, immune and other cell types. Feeder cells can be used to support the indifferentiated growth of stem cells. They provide an environment with several secreted molecules, adhesion molecules, and ECM proteins (Li et al., 2017). We cultured the ENS cells on a feeder-layer of the 3T3 lineage of mouse embryonic fibroblasts. On 3T3 feeder-layers, the glial cells did not underwent neuronal differentiation to the same extent, nor did enteric glial cells from adult or neonate mice (Figure 8). In this condition, fewer cells from adult mice were positive for both GFAP and βIIItubulin after 7 days in culture, comprising only 40.0 ± 9.8% (Figures 8A,C), whereas in the control they comprised 73.8 ± 12.9%, as we showed previously (Figure 1H); and the cells expressing only GFAP comprised 53.8 ± 10.8%, against 17.6 ± 14.0% in the control cultures. To determine whether soluble factors released by 3T3 could play a role in this differentiation-inhibition effect, we treated the enteric neural cells with the 3T3-conditioned medium (3T3-CM) (Figure 8B). No significant inhibition was observed compared to the control condition and 67.3 ± 14.2% were double-labeled cells (Figures 8B,C).

Neonate enteric glial cells cultured on the 3T3 feeder-layer also had their differentiation potential inhibited (Figure 8D). Cells double-labeled for GFAP+βIIItubulin+ comprised only 32.8 ± 9.9% after 6 days of co-culture (Figure 8F), while in the control they comprised 89.7 ± 7.4%, as mentioned above (Figure 2D). Cells expressing only GFAP comprised 53.3 ± 12.6%, and in the control comprised 2.8 ± 1.6%. With 3T3-CM the inhibition was not as marked as on the 3T3 substrate, but still this condition led to a significant reduction of cells expressing both the glial and neuronal markers (Figure 8E). 3T3-CM reduced the percentage of GFAP+βIIItubulin+ cells to 68.8 ± 7.2% after 6 days in culture (Figure 8F).

### Laminin Is Expressed by Basement Membranes of the Muscular and Epithelial Cells of the Gut and Contact With It Reduces the Number of Enteric Glial Cells in Neuronal Differentiation Process *in vitro*

We observed that 3T3-CM did not significantly regulate the neurogenesis of adult enteric glial cells. Another possibility is that this effect was produced by the physical contact of enteric glial cells with 3T3 and the deposited proteins produced by this cell lineage.

One must consider the role of laminin, a protein of the ECM, that similarly to the fibroblast feeder-layers, may support the undifferentiated growth of some cell types, such as ES cells (Miyazaki et al., 2008). Accordingly, we also analyzed the expression of the ECM proteins laminin and fibronectin by the 3T3 fibroblasts. Our results showed that 3T3 cells expressed laminin and fibronectin (Supplementary Figure S2). We observed some neuronal and glial projections that followed the same pathway designated by laminin deposition (data not shown).

**FIGURE 9:**
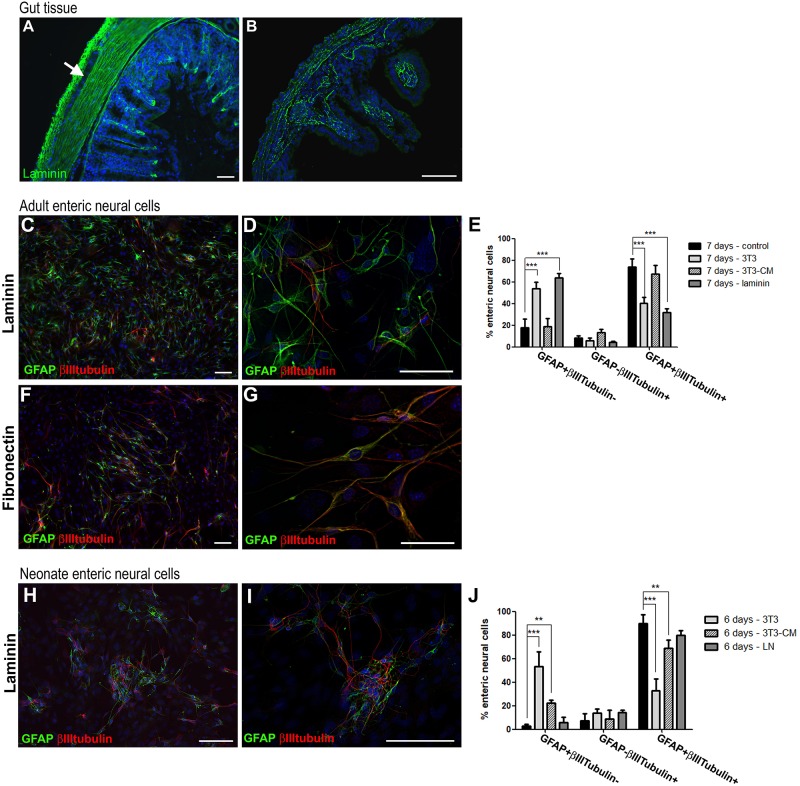
Culture of adult mouse enteric glial cells on laminin substrate inhibits neurogenesis. Analysis by immunostaining for GFAP and βIII-tubulin. **(A,B)** Muscle layers of adult mouse colon expressed high amounts of laminin **(A)**, and muscle layers from neonate mice showed sparse deposition of laminin **(B)**. Laminin expression can also be observed on basement membranes of adult and neonate mucosal layers. **(C,D)** Adult mouse enteric glial cells cultured on laminin substrate. **(E)** Graph shows the data of Figure 8C, plus the culture condition on laminin. On laminin, fewer cells expressed both GFAP and βIIItubulin, at a similar rate found in the co-culture with 3T3 fibroblasts. At the same time, a much larger number of cells expressed only GFAP. The results represent the mean of 3 independent experiments. ^∗∗∗^*P* < 0.001. **(F,G)** Adult enteric glial cells cultured on fibronectin substrate. As in the control condition, most of the cells expressed both GFAP and βIIItubulin after 7 days in culture. **(H,I)** Neonate enteric glial cells cultured on laminin substrate. **(J)** Graph shows the data of Figure 8F, plus the culture condition on laminin. The number of cells expressing only GFAP or both GFAP and βIIItubulin did not change significantly on laminin. The results represent the mean of 3 independent experiments. ^∗∗^*P* < 0.01, ^∗∗∗^*P* < 0.001. Scale bars: 50 μm **(A,B,D,G)** and 100 μm **(C,F,H,I)**.

The expression of laminin and fibronectin by 3T3 suggests that contact with these ECM could be involved in the maintenance of enteric glia that do not undergo neuronal differentiation. The next step was to investigate how the basement membrane produced by the gut cells is related to the enteric neural cells. We performed immunofluorescence analyses of adult and neonate gut tissue. We observed that laminin composes the basement membranes of epithelial and muscular layers of mucosa and also of muscle cells of the circular and longitudinal layers (Figures 9A,B). Longitudinal and circular muscle layers of the adult gut express large amounts of laminin (Figure 9A), while the laminin network of muscle layers of the neonate mouse gut is still sparse, suggesting that the ganglion cells may not necessarily be in contact with laminin at that time (Figure 9B). ENS ganglionic cells seem to not express laminin (arrow at Figure 9A), but in the adult mouse they are in close contact with it, especially the cells composing the myenteric ganglia, located between the laminin layers.

Therefore, in order to understand the real function of the contact of enteric glia with laminin in the differentiation process, we prepared a poly-laminin substrate (Freire et al., 2012) to culture the enteric neural cells of adult (Figures 9C,D) and neonate (Figures 9H,I) mice. The fibronectin substrate was also used in the ENS cell culture (Figures 9F,G). The number of enteric glial cells from adult mice who acquired the expression of βIIItubulin decreased markedly during the 7 days of culture. GFAP+βIIItubulin+ cells comprised 31.8 ± 6.1%, while in the absence of the substrate they comprised 73.8 ± 12.9% (Figure 9E). Cells expressing only GFAP comprised 17.6 ± 14.0% in the control and 63.3 ± 7.2% on laminin. When cultured on the fibronectin substrate, the neural cells showed a quite different morphology, with longer neural projections (Figures 9F,G), and the cell culture contained a markedly larger number of cells, suggesting a role in proliferation. However, the fibronectin substrate showed no observable function in the neuronal differentiation process, and the cells acquired βIIItubulin expression, as occurred in the control conditions. These observations reinforce the specific role of laminin in this function of inhibiting enteric glia differentiation.

For enteric glial cells of neonate mice, on the other hand, neurogenesis was not significantly inhibited by contact with the laminin substrate (Figures 9H,I). Although the GFAP+βIIItubulin+ double-labeled cells showed a lower fluorescence intensity than the control cells, they were still very numerous, comprising 79.6 ± 4.0% of the neural cells found in cultures at 6 days. Cells expressing only GFAP comprised only 5.8 ± 4.5% in this condition (Figure 9J).

Our *in vitro* results strongly suggest that the cell microenvironment acts to maintain the differentiation potential of enteric glia, and that the laminin deposits located around the ganglia are a newly recognized factor in controlling the process of differentiation of adult enteric glia into neurons.

## Discussion

By culturing enteric glial cells *in vitro*, we observed that after a few days most of the enteric neural cells naturally showed both the glial marker GFAP and the neuronal marker βIIItubulin. The proportion of these double-labeled cells increased gradually in the first days of culture, reaching 73.8 ± 12.9% of the neural cells in the 7-day adult cell cultures and 89.7 ± 7.4% in the 6-day neonatal cell cultures (Figures 1H, 2D).

Some cells double-labeled for GFAP and βIIItubulin were in a proliferative state, and others were not. These observations together with the fact that the cells express the glial and neuronal markers in the same exact moment, indicate that the glial cells were in the process of transdifferentiating into neurons, which is in agreement with a previous study that observed transdifferentiation of enteric glial cells *in vivo* in cases of colitis (Belkind-Gerson et al., 2017). In that study, with *in vivo* experiments, the Sox2-positive enteric glial cells also showed double labeling with the HuC/D neuronal marker during the differentiation process, and were negative for RET, which is typically present in neurons and progenitor cells. Laranjeira et al. (2011) demonstrated that the glial marker GFAP was lost before the acquisition of the βIIItubulin marker. While in our experiments a transdifferentiation process seemed to occur without the need for proliferation, that study indicated dedifferentiation followed by differentiation initiated by Mash1, at least in part of the neurons.

The ability of glial cells to become neurons has also been observed in the central nervous system by some studies. It has already been shown that astrocytes of the subventricular zone of the adult mice brain give rise to neurons of the olfactory bulb. They are also able to generate immature progenitors and neuroblasts in the region, after elimination of neurons and progenitors with antimitotic treatment, and form multipotent neurospheres *in vitro* (Doetsch et al., 1999). Astrocytes of the cerebral cortex, spinal cord, cerebellum and subependymal zone of neonatal mice of up to 2 weeks form neurospheres that give rise to both neurons and glia. In adult mice this capacity was observed only in the astrocytes of the subependymal zone (Laywell et al., 2000). Cells differentiated from neurospheres generated from cells of the subependymal zone or cerebellar cortex of postnatal mice also present from 3 days in culture the expression of both GFAP and βIIItubulin markers (Laywell et al., 2005).

We also analyzed the presence of Sox10 in those glial cells that initiated neuronal differentiation with βIIItubulin expression. During development, neuronal differentiation of the enteric neural crest involves the inhibition of Sox10, which continues to be expressed only in glial cells. Interestingly, a distinct majority of GFAP+βIIItubulin+ cells continued to express Sox10. The GFAP+βIIItubulin+Sox10- or GFAP-βIIItubulin+Sox10+ cells were rare.

Our RT-PCR experiments reinforce this sequential program of differentiation. Interestingly, at 3 days, when βIIItubulin-positive cells correspond to only 13.8% of the neural cells (and 8.8% of total cell number, including non-neural cells), before overt morphological differentiation, we found a high RNA expression of βIIItubulin when compared with GFAP expression (97.7% of the neural cells and 62.7% of total cell number), which suggest that neurogenesis starts *in vitro* before the production of βIIItubulin protein by the glial cells.

Recently, Kulkarni et al. (2017) suggested that neural progenitors exist in the gut of healthy adult mice, and replace most of the enteric neurons lost by apoptosis every 2 weeks. According to the authors, these progenitor cells express nestin, but not Sox10, thus composing a nestin+Sox10-cell population different from the sox10-positive enteric glial cells. In contrast, Joseph et al. (2011), characterizing the enteric glial cells isolated from the myenteric plexus of the mouse colon by CD49b expression, showed that 98% of them expressed Sox10 and 93% expressed nestin, making it clear that most glial cells show both markers, contrary to the findings of Kulkarni et al. (2017). Our results showed that nestin was expressed *in vitro* by most enteric glial cells (Supplementary Figure S3), which also expressed GFAP and Sox10 (Figures 1I–J’). In addition, a study in mice, that received pulses of thymidine analogs at regular intervals, showed that enteric neurons arise from E8 until the first 3 weeks of life only (Pham et al., 1991).

In long-term cultures of 14 and 21 days it was possible to detect the presence of HuC/D and peripherin (Figure 4), neuronal markers that arise later than βIIItubulin. This was not observed in the 7-day cultures, where only neurons from the primary culture were positive for these markers (data not shown). In later cultures, we readily observed the presence of cells expressing only the βIIItubulin marker, indicating that by continuing the neurogenesis process, the original glial cells lose the GFAP expression (neurons arising to the cell culture directly from intestinal tissue, that do not appear from the *in vitro* differentiation of enteric glial cells, are mostly lost in the 7-day passage).

The markers analyzed *in vitro* were also evaluated directly in the intestinal tissue, in the colon and/or ileum of adult mice. We did not find co-localization of the glial and neuronal markers when we analyzed the myenteric plexus ganglia adhered to the longitudinal muscular layer (LMMP), confirming that *in vivo* these markers are not commonly co-expressed.

It is known that stem cells cultured on fibroblasts monolayers can maintain their undifferentiated growth (Domogatskaya et al., 2008; Rodin et al., 2010). Our results showed that a co-culture system with 3T3 fibroblasts also restricted the differentiation of enteric glial cells from neonatal mice, where only 32.8 ± 9.9% expressed GFAP and βIIItubulin; and from adults, where only 40.0 ± 9.8% were double-labeled. In this way this culture condition does seem to more closely resemble the condition of enteric glial cells *in vivo*.

According to Gershon (2011), the disruption of contact between cells may initiate neurogenesis from precursors that express enteric glial markers. However, our data showed that even after the dissociation of the neural cells performed in the culture protocol, enteria glial cells showed a significantly lower rate of differentiation when they were dispersed on the 3T3 monolayer. What property of 3T3 fibroblasts might be responsible for causing the smallest number of cells in neurogenesis? One might consider the possibility of an effect of secreted soluble growth factors. However, we did not observe a significant change in the differentiation rate when we cultured the adult ENS cells in the presence of conditioned medium from 3T3 fibroblasts. Only co-cultured enteric glia from newborn mice showed a significant reduction in the number of cells expressing both GFAP and βIIItubulin, although smaller than the inhibition promoted by the substrate of 3T3 fibroblasts.

Connective tissue cells typically secrete large amounts of ECM proteins. Another reason why 3T3 reduced the differentiation in relation to the control, might be through the action of secreted proteins that compose the ECM. In fact, the fibroblast lineage used secretes the ECM protein laminin, as well as fibronectin (Supplementary Figure S2). When we analyzed cells grown on the laminin substrate, we found that this condition caused greater inhibition of the neuronal differentiation of enteric glia from adult mice, reducing the number of GFAP+βIIItubulin+ cells by more than 50% after 7 days (from 73.8 to 31.8%). In addition, we did not observe inhibition of neuronal differentiation when the enteric glial cells of adult individuals were cultured on the fibronectin substrate, which serves as a control for the specific role of laminin. Interestingly, the inhibition of the neurogenesis of enteric glia from neonate mice on laminin substrate was not significant, decreasing only about11.2% the percentage of GFAP + βIIItubulin + cells (from 89.7 to 79.6%). Our findings differ from those previously found, where neither laminin nor other ECM molecules investigated were able to inhibit the neuronal fate of neural progenitor cells from the adult rabbit gut (Raghavan et al., 2013). These results allow us to hypothesize that 3T3 promotes inhibition of neuronal differentiation of enteric glia by different mechanisms in cells from adult or neonate mice.

This study suggests that the rupture of contact between the adult enteric glial cells and laminin composing the ECM produced by the cells located around them is able to trigger neurogenesis. Given this first step, it is important that further studies look at the intracellular molecular processes that follow. Results obtained with *ex vivo* culture of longitudinal muscle and myenteric plexus tissue suggest that ENS neurogenesis can occur in a PTEN-dependent manner (Becker et al., 2013). Liu et al. (2009) demonstrated that it is possible to induce neurogenesis in myenteric plexus ganglia through activation of the serotonin receptor with the 5-hydroxytryptamine agonist 4 (5-HT 4). It is possible that these mechanisms are involved in the neurogenesis of enteric glial cells when they lose their contact with laminin. Understanding how the neurogenic potential of enteric glia is activated is the first step in thinking about inducing the generation of new neurons in damaged areas, which could possibly lead to ENS regeneration.

Taking the above into consideration, our results reinforce the importance of the microenvironment molecules in inhibiting the neuronal differentiation of enteric glia. Culturing these cells in a more similar condition to the *in vivo* environment appears to hold the glial phenotype and to keep inhibited the neurogenic potential of the enteric glial cells, suggesting that, in normal conditions and with appropriate interactions with the microenvironment molecules, enteric glia can act in its glial functions without undergoing neuronal differentiation, and only severe disruption can alter significantly the balance of soluble and ECM molecules and trigger this phenomenon.

## Data Availability

All datasets generated for this study are included in the manuscript and/or the Supplementary Files.

## Ethics Statement

Newborn (P0 or P1) and adult (P90–P120) male Swiss mice were used. This research project was approved by the Animal Use Ethics Committee of the Centro de Ciências da Saúde-Universidade Federal do Rio de Janeiro (CCS-UFRJ) (protocol no. 129/16).

## Author Contributions

JC-A was responsible for the overall project conception and design. JC-A, LC, and VM-N supervised the project. CV, JC, FS, and JC-A performed the experiments. CV and JC-A wrote the manuscript.

## Conflict of Interest Statement

The authors declare that the research was conducted in the absence of any commercial or financial relationships that could be construed as a potential conflict of interest.

## Supplementary Material

FIGURE S1 Culture of adult mouse ENS cells show the presence of myofibroblats. Cells at day 7 in cell culture on laminin substrate. Scale bar: 25 μm.

FIGURE S2 Cells from the 3T3 fibroblast lineage express laminin **(A)** and fibronectin **(B)**. Scale bar: 50 μm.

FIGURE S3 Enteric glial cells from adult mice express Nestin *in vitro.* **(A)** At day 3, Nestin expression was observed in most cells. **(B)** Cells at day 7 in cell culture on laminin substrate. Scale bar: 50 μm.
