# Supplementary figures and images for: Laminin and Environmental Cues Act in the Inhibition of the Neuronal Differentiation of Enteric Glia in vitro
Source: Front Neurosci. 2019 Sep 3;13:914. doi: 10.3389/fnins.2019.00914 (PMC6733987; doi:10.3389/fnins.2019.00914)

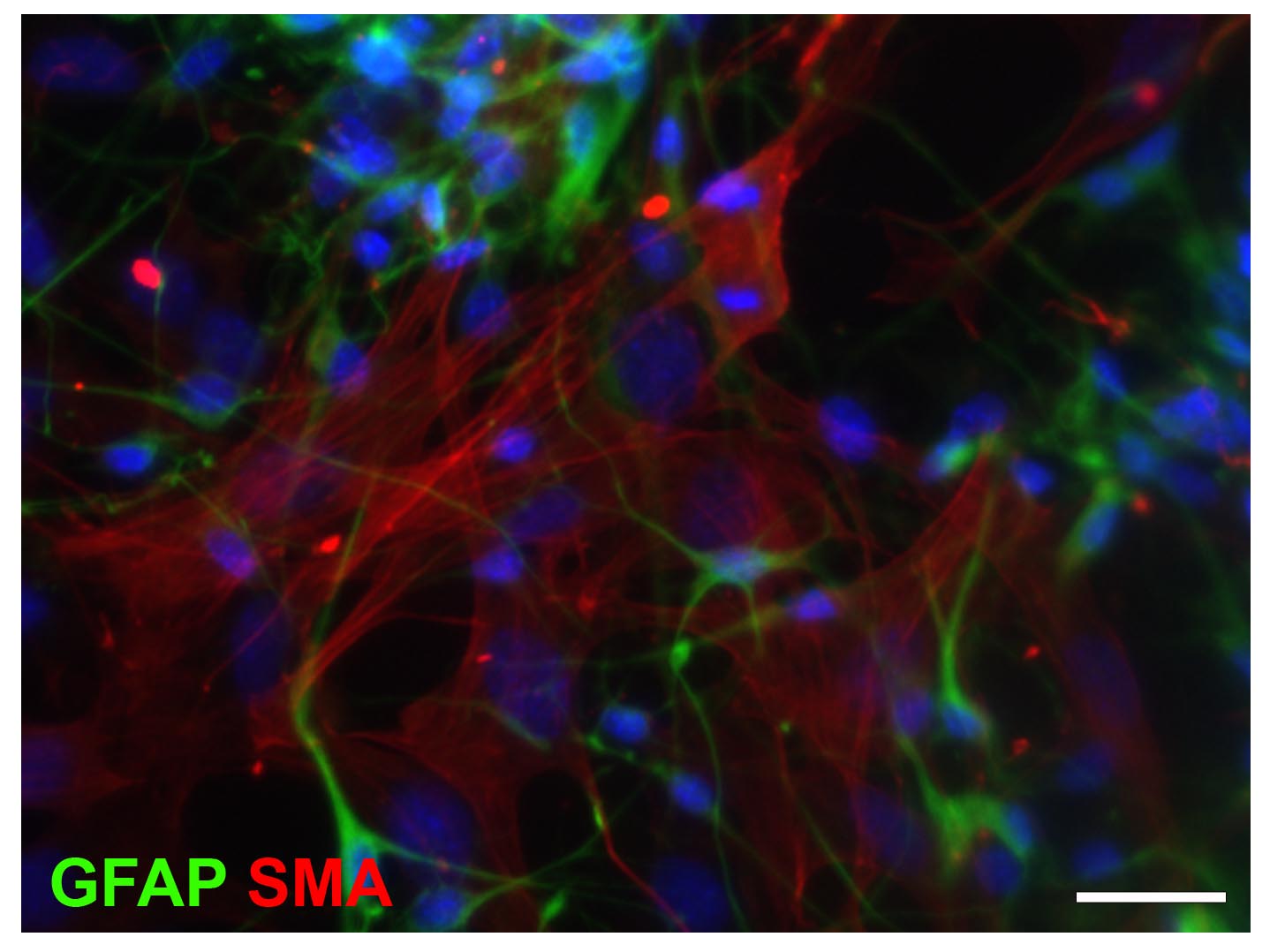

Supplement: FIGURE S1 — Culture of adult mouse ENS cells show the presence of myofibroblats. Cells at day 7 in cell culture on laminin substrate. Scale bar: 25 μm. [file Image_1.JPEG]

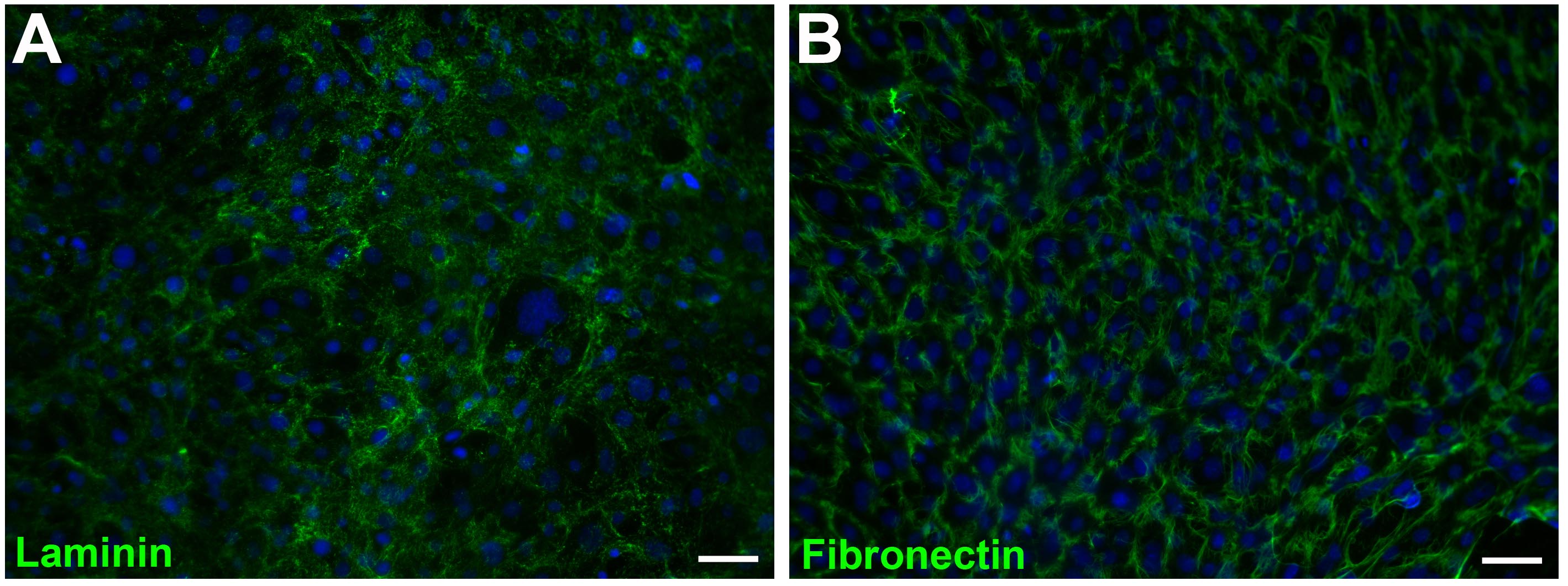

Supplement: FIGURE S2 — Cells from the 3T3 fibroblast lineage express laminin (A) and fibronectin (B). Scale bar: 50 μm. [file Image_2.JPEG]

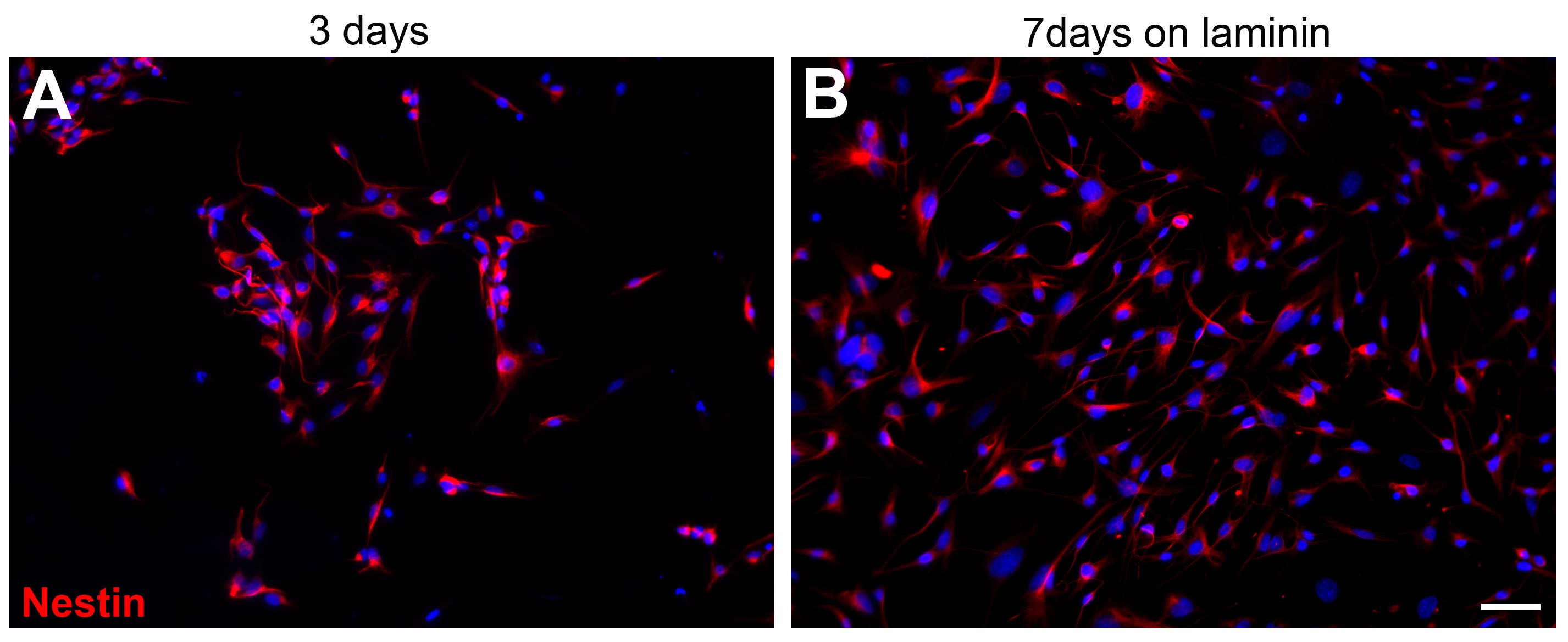

Supplement: FIGURE S3 — Enteric glial cells from adult mice express Nestin in vitro. (A) At day 3, Nestin expression was observed in most cells. (B) Cells at day 7 in cell culture on laminin substrate. Scale bar: 50 μm. [file Image_3.JPEG]
